# Entomopathogenic Potential of *Simplicillium lanosoniveum* Native Strain in Suppressing Invasive Whitefly, *Aleurodicus* *rugioperculatus* Martin (Hemiptera: Aleyrodidae), Infesting Coconut

**DOI:** 10.3390/jof7110964

**Published:** 2021-11-12

**Authors:** Maruthakasi Sujithra, Hanumanthappa Veerappa Prathibha, Manikappa Rajkumar, Govindharaj Guru-Pirasanna-Pandi, Sengottayan Senthil-Nathan, Vinayaka Hegde

**Affiliations:** 1 Division of Crop Protection, Central Plantation Crop Research Institute, ICAR, Kasaragod 671124, India; prathibhacpcri@gmail.com (H.V.P.); rajkumarcpcri@gmail.com (M.R.); hegdev64@gmail.com (V.H.); 2 Division of Crop Protection, National Rice Research Institute, ICAR, Cuttack 753006, India; 3 Division of Biopesticides and Environmental Toxicology, Sri Paramakalyani Centre for Excellence in Environmental Sciences, Manonmaniam Sundaranar University, Tirunelveli 627412, India

**Keywords:** plantation, environmental safety, whitefly, invasive pest, mycoinsecticide, entomopathogenic fungi

## Abstract

In 2016, infestation of an exotic polyphagous pest, the rugose spiraling whitefly (RSW), *Aleurodicus rugioperculatus* Martin (Hemiptera: Aleyrodidae), was documented on coconut for the first time in India. Instantaneously, RSW has garnered wide attention owing to its damage severity and rapid spread across the coconut-growing regions of the country. Hence, an attempt was made to devise a sustainable integrated pest management (IPM) module using biological control agents as a mainstay component. The present study documented the identification and characterization of a potential entomopathogenic fungal isolate for the management of RSW. An entomopathogenic fungus isolated from nymphal cadavers of RSW was identified as *Simplicillium lanosoniveum* based on morphological and phylogenetic analyses. A gradient of five conidial concentrations (1 × 10^4^, 1 × 10^5^, 1 × 10^6^, 1 × 10^7^ and 1 × 10^8^ conidia/mL) of the *S.* *lanosoniveum* were tested against eggs, first instars, second to third instars and pupae of RSW. Results revealed that *S.* *lanosoniveum* is highly virulent to all developmental stages of RSW by causing mortality rates of 95.20%, 87.33%, 85.38% and 72.85%, in eggs, initial, middle and later instar nymphs of RSW, respectively, at the highest tested concentration (1 × 10^8^ conidia/mL) at seven days after exposure. The LC_50_ and LT_50_ values of *S.* *lanosoniveum* were 4.72 × 10^4^, 4.94 × 10^4^, 5.11 × 10^5^, 5.92 × 10^5^ conidia/mL and 4.27, 4.86, 4.56, 5.89 days against eggs, initial, middle and later instar nymphs of RSW, respectively. Further, preliminary field trials with *S.* *lanosoniveum* strain at 1 × 10^8^ conidia/mL exhibited a significant reduction in the egg and nymphal population by 57.8% and 56.3%, respectively. This report thus demonstrated that the newly isolated *S.* *lanosoniveum* is an effective pathogen at suppressing all the developmental stages of RSW. This is the first record of *S.* *lanosoniveum* infecting RSW, and it has a great potential to be developed as a mycoinsecticide.

## 1. Introduction

Rugose Spiraling Whitefly (RSW), *Aleurodicus rugioperculatus* Martin (Hemiptera: Aleyrodidae) is an introduced pest, whose infestation on coconut was reported for the first time from Pollachi, Tamil Nadu (10.6609° N, 77.0048° E) and Palakkad, Kerala (10.7867° N, 76.6548° E) state in India [1]. RSW infestation on coconut was first described from Belize [2], and Central America is believed to be the centre of origin for the pest. RSW is a highly polyphagaous pest with more than 118 hosts belonging to 43 diverse plant families. Among the hosts, palms constitute 22%, followed by *Bursera simaruba* (16%), *Calophyllum* spp., (10%), avocado (9%), black olive (4%), and mango varieties (3%) [3]. RSW has already been identified as a serious threat to the cultivation of coconut palms and other *Arecaceae* species in Florida [4,5]. Initially, 17 plant species (belonging to 11 families) were recorded as preferred hosts for RSW after the pest reached South Kerala [6]. Subsequently, the incidence of RSW was reported in the states of Kerala, Karnataka, Andhra Pradesh, Assam, Goa, West Bengal, Maharashtra and Gujarat on several crops such as coconut, banana, sapota, corn, oil palm, mango, cashew and many other ornamental plants [7,8]. The availability of a wide range of host plants, combined with congenial climatic conditions, favored their successful establishment in the areas of invasion. Both the nymphs and adults of RSW directly suck the phloem sap of the leaf and secrete honey dew which encourages the growth of sooty mould on the leaf surface that eventually reduces the photosynthesis in the affected palms. Currently, RSW has become a regular pest of coconut, warranting effective integrated pest management (IPM) strategies.

Among the various biocontrol agents, entomopathogenic fungi (EPF) have been found to be successfully developed as mycopesticides in managing piercing—sucking groups of insects including whiteflies. Whiteflies are known to be infected by more than 20 species of entomopathogenic fungi, of which some of the widely studied fungi are *Beauveria bassiana* (Vuillemin)*,* *Akanthomyces lecanii* (Zimm.) and *Cordyceps fumosorosea* (Wize) [9,10,11,12]. EPFs are considered as potent alternatives for whitefly management because of their unique ability to invade the host insects directly by penetrating through the integument without the concern of resistance development [13]. 

Severe infestation of RSW in palm oil resulted in a reduction of 20–25% bunch yield at Andhra Pradesh [14]. Likewise, RSW has become a serious predicament for coconut growers, hence efforts were made to identify the indigenous potential biological control associated with the invasive pest. The fungal strain was identified and characterized based on morphological descriptions coupled with molecular analysis. To the best of our knowledge, this is the first report of the occurrence of entomopathogenic fungi, *S.* *lanosoniveum* (Cordycipitaceae: Hypocreales) on RSW. Furthermore, the efficacy of the indigenous fungal strain was investigated under laboratory and field bioassays in order to develop a biopesticide for the management of this emerging pest. 

## 2. Materials and Methods

### 2.1. Insect Cultures

RSW adults were collected from infested coconut gardens of the research farm belonging to ICAR—Central Plantation Crops Research Institute (ICAR—CPCRI), Kasaragod, Kerala, India (12°30′ N latitude, 75°00′ E longitude and 10.7 m). RSW culture was reared on coconut seedlings raised in pots placed in the greenhouse with natural photoperiodic conditions. Different developmental stages of RSW such as eggs, first instar, second instar, third instar and fourth instar nymphs (pupae) were collected from the reared culture and were used for bioassay studies. 

### 2.2. Fungal Isolation

RSW nymphal cadavers (*n* = 4) fully covered with fungal mycelia under natural growth conditions were collected from a coconut garden at ICAR—CPCRI, Kasaragod (12°30′ N latitude, 75°00′ E longitude and 10.7 m). Sterile needles were used to isolate mycelia or conidia from the cuticles of nymphal cadavers transferred to a Petri plate containing Potato Dextrose Agar (PDA) medium and cultured at 26 ± 1 °C and 60% RH for 7–10 days in a biochemical incubator (BOD). In order to isolate the pure culture, the hyphal tip from a small fungal colony was transferred to PDA slants and incubated at 26 ± 1 °C and 60% RH. The morpho-taxonomic characteristics of the fungus were identified based on conidia—forming mycelia and conidial structure [15,16]. 

### 2.3. Identification of the Fungus

#### 2.3.1. Morphological Identification 

Morphological identification of the fungal isolate was recorded on PDA medium incubated at 26 ± 1 °C and 60% RH for seven to ten days. The micro—morphological features were recorded under a compound microscope (Nikon Eclipse 80i) attached with a digital camera. The identification of the fungus was further confirmed with the help of Fungal Identification Services, Agharkar Research Institute (ARI), Pune, Maharashtra, India. 

#### 2.3.2. Genetic Identification

Total genomic DNA was extracted from five-day old single spore cultures of fungus grown in 100 mL Potato Dextrose broth. After vacuum filtration, the mycelia were washed with sterilized distilled water and powdered using liquid nitrogen with the help of a mortar and pestle. The DNA was extracted from powdered preparations using DNeasy Plant Mini Kit (Qiagen, Valencia, CA, USA) following the standard manufacturer’s instructions. The fungal ITS region was amplified using the primers: ITS4 (5′-TCCTCCGCTTATTGATATGC-3′) and ITS6 (5′-GGAAGTAAAAGTCGTAACAAGG-3′) [17]. Two more nuclear genes *viz.*, elongation factor-1 alpha (*TEF1*-α) and RNA polymerase II largest subunit 1 (*RPB1*) of the ribosomal RNA gene were used in addition to confirm the identification the species level. The DNA was amplified using specific primers of the *TEF1*-α (983F 5′ GCYCCYGGHCAYCGTGAYTTYAT 3′ 2218R 5′ ATGACACCRACRGCRACRGTYTG and *RPB1* (CRPB1 5′ CCWGGYTTYATCAAGAARGT 3′ and CRPB1 A 5′ CAYCCWGGYTTYATCAAGAA 3′ RPB1Cr 5′ CCNGCDATNTCRTTRTCCATRTA 3′ [18]. The resultant PCR products were excised and purified using a Qiagen Gel Extraction Kit (Qiagen India) following the standard protocol and were sequenced at Agrigenome, Cochin, India. The generated sequences were submitted to NCBI GenBank (Accession numbers: ITS = MK463992, *TEF1*-α = MW893683 and *RPB1* = MW836952) and used for multi gene phylogenetic analysis. The sequences were used as query to perform BLAST search (http://blast.ncbi.nlm.nih.gov/ (accessed on 8 April 2021), and the sequence similarity studies were performed using ClustalX (1.81). The phylogenetic tree of *S.* *lanosoniveum* was constructed following maximum likelihood (ML) method using MEGA X software [19].

### 2.4. Virulence Bioassays

#### 2.4.1. Conidial Preparation

The fungal culture was inoculated in Potato Dextrose Broth (PDB) and incubated at 26 ± 1 °C and 60% RH on a shaker for 14 days. The mycelia mat along with conidia was thoroughly mixed in a mixer grinder, and the suspension was filtered through three layers of muslin cloth, in order to get a hyphal—free conidial suspension. Conidial suspensions were prepared in sterile distilled water containing 0.01% Tween 80 and the concentration of the suspension was adjusted to different conidial concentrations using Neubauer’s improved haemocytometer under a Nikon eclipse 80i compound microscope. Five different spore concentrations ranging from 1 × 10^4^ to 1 × 10^8^ conidial concentration/mL were tested against different developmental stages of RSW. 

#### 2.4.2. Experimental Design

Coconut leaf bits containing 35–40 different developmental stages of RSW were collected from the greenhouse culture. Thirty leaf bits (10 cm each) were gently dipped in freshly prepared respective conidial suspensions for 20 s and placed separately on a Petri plate (14 × 2.5 cm) over sterilized wet tissue paper. Sterile distilled water containing 0.01% Tween 80 alone was used as the control. The experiment had five replications for each concentration, and the whole bioassay was repeated twice. Prior to conidial spray, the number of various developmental stages of RSW on each coconut leaf bit was counted to maintain a uniform population across the treatments. The mortality data were recorded by counting dead cadavers and developmental stages with fungal infection at 24 h intervals up to seven days, and the data was subjected to Abbott’s formula [20] for calculating corrected mortality. For time mortality studies, the higher concentration at 1 × 10^8^ spores/mL were used to determine LT_50_ of *S. lanosoniveum* against RSW developmental stages.

### 2.5. Field Virulence of S. lanosoniveum

Two field trials using *S.* *lanosoniveum* carried out during November 2019 and January 2020, respectively, in the RSW infested coconut gardens of ICAR—CPCRI, Kasaragod, Kerala (average temperature 22–33 °C, 60–73% RH during December 2019 and average temperature 20.6–32.4 °C, 59–71% RH during January 2020, respectively). The conidial suspension of the fungal culture (1 × 10^8^ conidia/mL) was prepared in sterile aqueous solution containing 0.01% Tween 80. The spore suspension of 500 mL was sprayed thoroughly on the dwarf cultivars of each coconut tree (variety; Chowghat Orange Dwarf (COD), *n* = 20) which were naturally infested with different developmental stages of RSW through handheld sprayer. Prior to fungal spray, the numbers of developmental stages of RSW per leaflet (5 leaflets per palm) were counted under a Nikon Stereozoom microscope (SMZ 800 N). Twenty dwarf palms sprayed with sterile aqueous solution containing 0.01% Tween 80 were maintained as control. Mortality data ware recorded from the treated and control plants after 14 days of spray. The experiment was replicated thrice and field bioassays were conducted twice at different time intervals. Developmental stages with *S. lanosoniveum* fungal infection recorded using a Nikon Stereozoom microscope (SMZ 800 N) and the percent mortality was calculated. 

### 2.6. Statistical Analysis

Conidial concentration, time and their interactive effects on mortality per cent data were analyzed using PROC GLM (SAS version 9.3, SAS institute, 2011). Mortality data of different developmental stages of *A. rugioperculatus* were corrected using Abbott’s formula [20]. The treatment difference was evaluated using least significant difference (LSD) at *p* < 0.05. The dose and time dependent mortality studies to kill 50% of the population (LC_50_ and LT_50_) were calculated by probit analysis [21] using R software based on binomial GLM function [22,23]. Field efficacy data were assessed by Student’s *t*-test with a significance of difference at *p* < 0.01 [24]. 

## 3. Results 

### 3.1. Fungal Identification

#### 3.1.1. Morphological Identification

**Figure 1:**
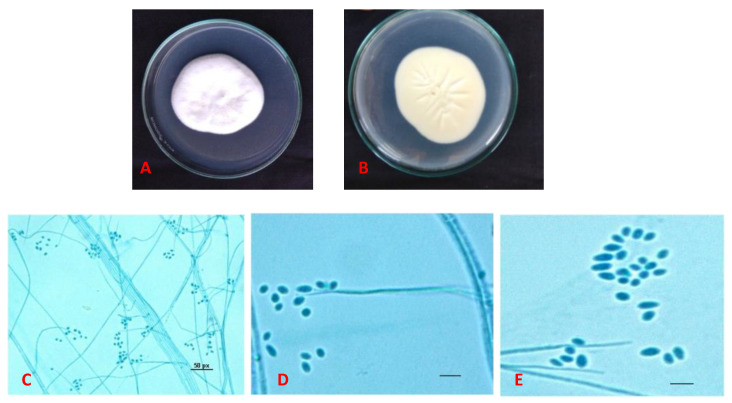
Morphological characters of *Simplicillium lanosoniveum.* (**A**,**B**) *Simplicillium lanosoniveum* cultured on PDA medium at 26 °C for 10 days (**A**) the front side (**B**) the reverse side (**C**,**D**) Conidiophores with conidiogenous cells (**E**) Conidia—cylindrical, oval-ellipsoidal (2–4 × 1–2.0 μm); scale bar (**D**,**E**); 50 µm.

Based on the morphological characters, the fungus isolated from the field collected nymphal cadavers of *A. rugioperculatus* was identified as *Simplicillium* *lanosoniveum* (Cordycipitaceae: Hypocreales). The fungal colonies were characterized on PDA medium as white, velvety with radial cracks and primrose yellow on the reverse side (Figure 1). Conidia was (2–4 × 1–2 μm), oval ellipsoidal, hyaline, smooth walled, adhering in globose to ellipsoidal head at the apex of phialides (Figure 1). 

#### 3.1.2. Genetic Identification

**Figure 2:**
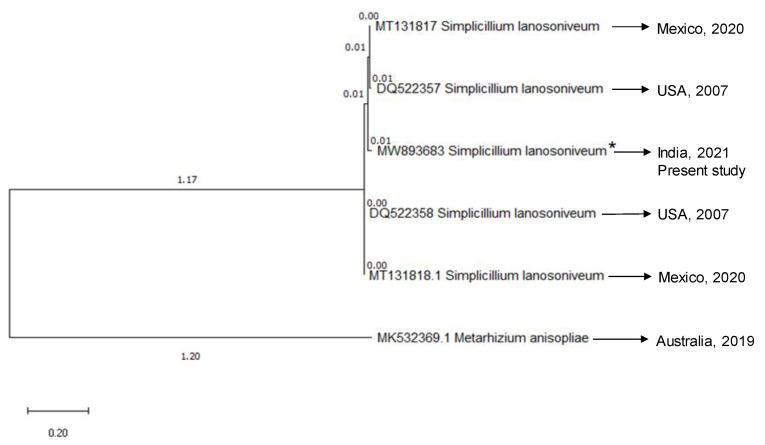
Phylogenetic tree based on maximum-likelihood analysis of combined ITS, b-tubulin and EF-1a sequences data. The sequence MW893683 (*) is the sequence generated in the present study and it branched with the retrieved sequences of *S. lanosoniveum*. *Metarhizium anisopliae* (MK532369.1) represented the outgroup.

The sequences of *Simplicillium* isolate generated in the present study have been deposited in GenBank with accession numbers: ITS = MK463992, *TEF1*-α = MW836952 and *RPB1* = MW893683. BLAST analysis of generated sequences exhibited 99% sequence similarity with *Simplicillium lanosoniveum* (DQ522406 and DQ522357). A phylogenetic tree of *Simplicillium* at species level was generated using maximum-likelihood (ML) analysis based on a combined data set of ITS, *TEF1*-α and *RPB1*. Multigene phylogenetic analysis showed that *Simplicillium* isolate (MK463992, MW836952 and MW893683) of the present study have a close phylogenetic affinity to *Simplicillium lanosoniveum* with strong bootstrap support (98–100%, Figure 2). 

### 3.2. Virulence Bioassay

**Figure 3:**
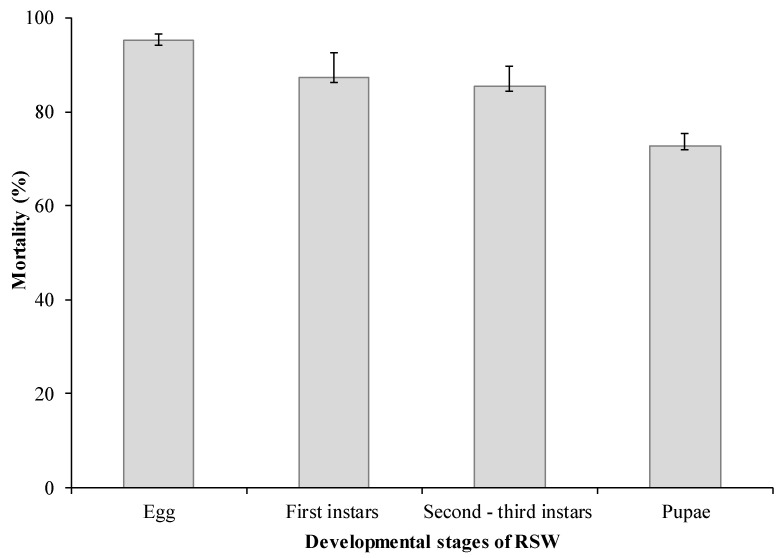
Pathogenecity of *S. lanosoniveum* tested at 1 × 10^8^ conidia/mL against different developmental stages of RSW. Data are mean ± SEM of three tests.

The pathogenicity of *S. lanosoniveum* was evaluated against different developmental stages of RSW. The fungal bioassay treatment resulted in the highest pathogenicity to the eggs, initial (first), middle (second—third) and later (fourth) instar nymphs of RSW with corrected mortality rates of 95.20%, 87.33%, 85.38% and 72.85%, respectively following the seventh day after treatment (DAT) at 1 × 10^8^ conidial concentrations/mL (Figure 3). 

**Figure 4:**
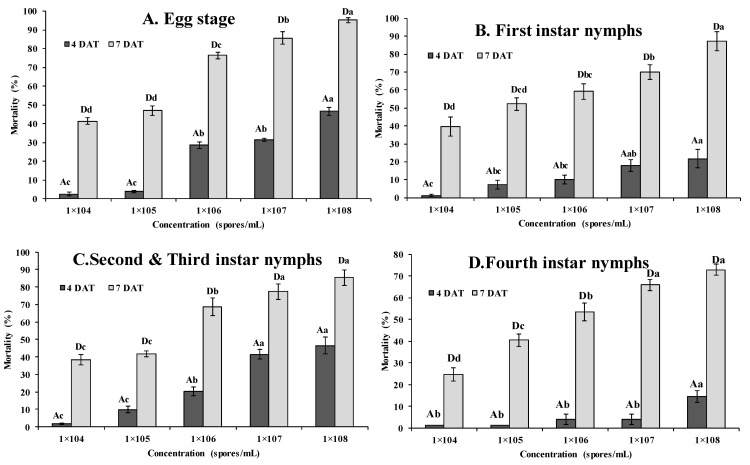
Mortality (% ± SE) of eggs (**A**), first instar nymphs (**B**), second and third instar nymphs (**C**), pupae (**D**) of *A. rugioperculatus* treated with fungus, *Simplicillium lanosoniveum*. Bars with different uppercase letters on the top of error bars indicate significant differences among the different days after treatment and with different lowercase letters indicate significant differences for different treatments i.e., concentration of fungus, *Simplicillium lanosoniveum*. (*p* < 0.05, Tukey’s test).

Different conidial concentration ranging from 1 × 10^4^ to 1 × 10^8^ conidia/mL treated against different developmental stages of RSW for evaluating its virulence are illustrated in Figure 4. The highest egg mortality of 95.20% was observed at higher conidial concentrations tested, (1 × 10^8^ conidia/mL) while low conidial concentration (1 × 10^4^ conidia/mL) exhibited mortality of 41.25% at 7 DAT (*F* = 40.46; df = 4,16; *p* < 0.0001) (Figure 4). Expectedly, egg mortality was found to increase with the increase in spore concentrations and exposure time (*F* = 52.61; df = 3,12; *p* < 0.0001). Likewise, first instars experienced highest mortality (87.33%) at conidial concentrations of 10^8^ conidia/mL (*F* = 8.18; df = 4,16; *p* < 0.0001) whereas lowest per cent mortality (39.64%) was observed at a dose of 10^4^ conidia/mL on 7 DAT (Figure 4). As observed in eggs, mortality in first instar nymphs increased significantly with increase in conidial concentration in each dose tested and with an increase in treatment period (*F* = 2.46; df = 12; *p* < 0.01). Similarly, on seven DAT, the highest mortality of 85.38% was observed in second to third instar nymphs treated with conidial concentrations of 10^8^ conidia/mL and the lowest concentration of 10^4^ conidia/mL recorded lowest mortality of 35.38% (*F* = 17.90; df = 4,16; *p* < 0.0001) (Figure 4). 

In case of pupae, highest mortality of 72.85% was documented with the dose of 10^8^ conidia/mL, whereas the lowest mortality of 24.76% was observed at the dose of 10^4^ conidia/mL on 7 DAT (*F* = 14.96; df = 4,16; *p* < 0.0001). The mean mortality in pupae ranged from 1.25% to 4.33% on 4 DAT when treated with conidial concentration of 10^4^ conidia/mL (*F* = 18.65; df = 3,12; *p* < 0.0001) and 10^8^ conidia/mL (*F* = 40.80; df = 3,12; *p* < 0.0001), respectively (Figure 4). A similar trend of increase in mortality with increase in conidial concentration of each dose tested and exposure time was also observed in pupae (*F* = 7.34; df = 12; *p* < 0.0001). Overall, the mortality rate at various developmental stages of RSW has significantly increased with an increase in the concentration of fungal conidia and the duration of the time following the exposure to the fungal treatments. In general, significant differences in the entomopathogenic pathogenicity of *S. lanosoniveum* against RSW were observed between conidial concentration (*F* = 215.29, df = 4; *p <* 0.0001), time interval (*F* = 803.91, df = 3, *p* < 0.0001) and stages of host insect (*F* = 145.64, df = 3; *p* < 0.0001). Significant interactions were observed between treatment*stage of host insect (*F* = 12.98, df =12, *p <* 0.0001), treatment*time (*F* = 6.55, df = 12, *p* < 0.0001), time*stage of host insect (*F* = 8.20, df = 9, *p* < 0.0001) and treatment*stage of host insect*time (*F* = 1.74, df = 36, *p* < 0.0001).

### 3.3. Determination of the Lethal Concentration (LC_50_) and Lethal Time (LT_50_)

Significant differences in the LC_50_ and LT_50_ values of *S. lanosoniveum* against different life stages of *A. rugioperculatus* were documented based on the dose—and time mortality response studies (Table 1). The median lethal concentration (LC_50_) values of *S. lanosoniveum* against eggs, first instars, second to third instarsand pupae of RSW were 4.72 × 10^4^, 4.94 × 10^4^, 5.11 × 10^5^, 5.92 × 10^5^ conidia/mL respectively. The LT_50_ values of *S. lanosoniveum* at the highest concentration (1 × 10^8^ spores/mL) on the corresponding developmental stages were 4.27, 4.86, 4.56, 5.89 days, respectively (Table 2). 

### 3.4. Field Virulence of Entomopathogenic Fungi

**Figure 5:**
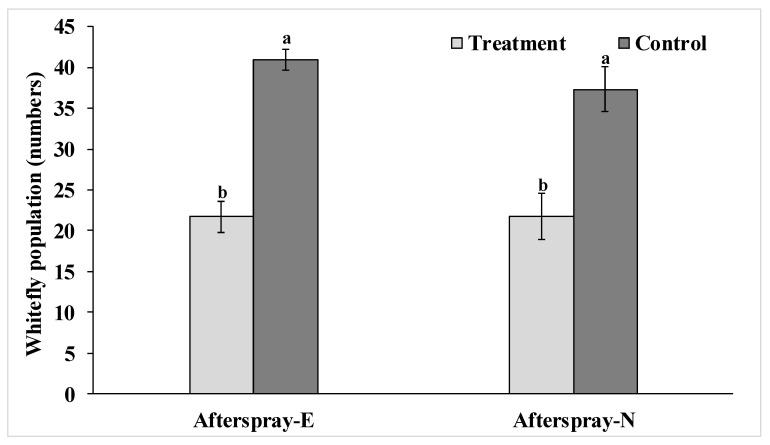
Field evaluation of *S. lanosoniveum* on the egg and nymphal population of RSW at 14 days after treatment (DAT). The different letters in Afterspray-E and Afterspray-N indicate differences between treatments. E—egg populations; N—nymphal populations.

Fourteen days after the spray of *S. lanosoniveum,* the mortality of eggs and nymphal stages of RSW was calculated. As shown in Figure 5, the population of eggs (*t* = 4.56; *p* < 0.01) and nymphs (*t* = 8.19; *p* < 0.01) of *A. rugioperculatus* were reduced by 57.8% and 56.3%, respectively at 14 DAT. Thus, the native fungi isolated and reported herein showed positive virulence by infecting RSW stages under open field conditions (Figure 5).

## 4. Discussion

The first incidence of the RSW was identified in India during the year 2016 following which it has spread widely and caused serious damages in larger areas of crop cultivation, particularly in coconut [6,7,8]. The adoption of management strategies using chemical pesticides is uneconomical and it also causes severe adverse effects on naturally existing biocontrol agents including *Encarsia guadeloupae* Viggiani (Hymenoptera: Aphelinidae) [7,8]. The conservation of natural enemy complexes and avoidance of insecticide spray are the major pest management strategies provided to the farmer’s against this invasive pest to avoid the ill effects of pesticide usage. In this context, utilization of entomopathogenic fungi as a means for biological control of RSW infestations is of significant importance [3,4,5,6]. However, exploration and identification of novel and virulent EPF strains adapted to particular localities is necessary for the development of effective mycoinsecticides. Efficacy of entomopathogenic fungi for managing the sucking insect pest population in the field conditions is well recognized [25] owing to their unique mode of action of penetrating the insect cuticle [26,27]. Among the EPFs, *Akanthomyces* species are widely utilized as commercial biological pesticides against whiteflies, thrips and aphids [28]. Fungicolous and entomopathogenic species of *Akanthomyces* were later placed in the genera *Simplicillium* and *Lecanicillium* based on systematic studies [16]. Although species of *Simplicillium* occurs in a broad range of ecological niches [16,29,30,31,32] but only few studies have reported the pathogenicity of *Simplicillium* sp. against insects [33,34,35,36,37,38,39,40]. In the present study, we report the identification of a novel isolate of entomopathogenic fungus, *S. lanosoniveum* isolated from RSW in the invaded region. Bioassay studies have revealed the potency of the isolate *S. lanosoniveum,* as it was found to be virulent against all the developmental stages of RSW. The ecological and economical value of *Simplicillium* sp. for its biocontrol and bioactive features were highlighted by many researchers [41,42,43]. *S.* *lanosoniveum* is a known phytopathogen, causing brown spots and lesions on flowers [44] or as a mycoparasite on soybean rust [45,46]. This species is also reported as a pathogen of aphids [33] and as an anti-*Trichomonas vaginalis* agent [47]. Other species of *Simplicillium* such as *S. chinense* acts as a biological control agent against plant parasitic nematodes [39,48]. *S. lamellicola* is known to suppress plant bacterial diseases and grey mould of tomato and ginseng [31,49]. Bioactive compounds with anti-fungal and anti-bacterial profiles and pharmaceutical exopolysaccharides were isolated from *S. lanosoniveum* [50,51,52,53]. Linear and cyclic peptides with anti-fungal, anti-bacterial and anti-viral properties were also discovered from the secondary metabolites of *S. lamellicola* and *S. obclavatum* [31,54]. The entomopathogenity of *S. lanosoniveum* was demonstrated against silkworms, and it was evident that virulence of the isolate was strong as that of *B. bassiana* [38]. The present study strain, *S. longisoniveum*, exhibited >70–80% mortality rates against RSW nymphs and pupae, while >90% mortality recorded for egg stage at 1 × 10^7^ and 1 × 10^8^ conidia/mL under laboratory bioassays on seven DAT. It was noticed that early instars are highly susceptible to this fungal strain than the late instars. Present study results are corroborating the earlier report of Nagasi et al. [55], where, *B. bassiana* was found most virulent against the first instar of silver leaf whitefly, *Bemisia argentifolii* (Bellows & Perring). Entomopathogenic fungi used as an effective biological control against in other whitefly species like, *B. argentifolii* and *Trialeurodes vaporariorum* (Westwood) [55,56,57]. Eyal et al. [58] reported that *B. bassiana* caused 52–98% mortality in *Bemisia tabaci* (Gennadius) at concentrations of 1–4 × 10^6^ conidia/mL. The present study observed increased mortality of different developmental stages of RSW with an increase in the conidial concentration and time of exposure. Likewise, Boopathi et al. [59] also observed that the application of *I. fumosorosea* was highly pathogenic to *A. dispersus* on cassava and exhibited differences in efficacy between 3 and 15 days after treatment. In the present study, the LC_50_ values and LT_50_ values of *S. lanosoniveum* against eggs, first instar, second to third instar, and pupae of RSW were 4.72 × 10^4^, 4.94 × 10^4^, 5.11 × 10^5^, 5.92 × 10^5^ conidia/mL and 4.27, 4.86, 4.56, 5.89 days, respectively. Similarly to present results, the LC_50_ values of *I. fumosorosea* for eggs, first—second, third and fourth instar nymphs of RSW were estimated to be 2.6 ×10^4^, 2.3 × 10^4^, 3.5 × 10^5^ and 9.5 × 10^5^ spores /mL respectively and the LT_50_ values were4.19, 3.44, 5.62 and 5.69, respectively for corresponding life stages for the concentration of 1 × 10^8^ conidia/mL tested [60]. Therefore, in terms of the pathogenicity bioassays, the LC_50_ and LT_50_ values of *S. lanosoniveum* exhibited significant biocontrol potential in the management of RSW under laboratory conditions. The results are comparable to the effects produced by the widely studied entomopathogenic fungi like *I. fumosorosea* [60]. However, *S. longisoniveum* at 1 × 10^8^ conidia/mL tested under field conditions caused 57.8% and 56.3% mortality in the egg and nymphal population of RSW on 14 DAT, which was less than that of laboratory efficacy. The reason for this reduction in efficacy in the field condition might be due to the involvement of abiotic factors, as field conditions are heterogenetic in nature compared to the homogenic environment of the laboratory. Earlier reports also suggested that environmental factors not only affect survival and virulence of entomopathogenic fungi, but also influence their host–pathogen interaction [61]. Temperature and relative humidity are the two most important environmental factors which greatly influence the fungal germination, infection, sporulation, survival and virulence of the entomopathogenic fungi [62,63,64]. In addition, the process of fungal infection is also governed by the microclimate of the insect cuticle [65]. However, the sensitivity of the isolate or specific strain varies with environmental factors [62] and the success of any entomopathogenic fungi is determined by its adaptability to the local or prevailing environmental conditions [66]. Therefore, it is imperative to ascertain the effect of temperature and RH on any new isolate before considering it for biocontrol of insect pests. The fungal isolate reported herein performed fairly well in reducing the whitefly population in the open field conditions where the prevailing temperature and relative humidity conditions could have played a crucial role in affecting its efficacy. To conclude, this is the first report of natural occurrence of *S. lanosoniveum* on RSW. This study identified a highly pathogenic fungal isolate, *S. lanosoniveum,* based on its morphology, molecular and phylogeny and demonstrated its pathogenicity against RSW under in vitro and field conditions. Hence, this study forms a foundation for the large scale development of a biocontrol agent to suppress the spread of RSW. However, further studies on its compatibility with natural enemies for field applications are required. Efforts are also in progress to develop formulations with high virulent stability to sustain its field efficacy.

## Figures and Tables

**Table 1:** Lethal concentration value of *S. lanosoniveum* against different developmental stages of RSW.

Developmental Stages of RSW	n ^†^	X^2^ (DF) ^‡^	Slope	LC_50_ *	LC_50_ Confidence Interval		LC_90_	LC_90_ Confidence Interval	
					Lower	Upper		Lower	Upper
Egg	958	11.20 (3)	6.14 ± 0.49	4.723 × 10^4 a^	3.799 × 10^3^	4.313 × 10^5^	7.706 × 10^7 a^	6.864 × 10^6^	9.560 × 10^9^
First instar	387	03.55 (3)	4.56 ± 0.69	4.946 × 10^4 ab^	4.270 × 10^4^	5.434 × 10^5^	10.122 × 10^7 a^	8.345 × 10^6^	15.99 × 10^9^
Second-third instar	247	09.89 (3)	5.03 ± 0.55	5.109 × 10^5 ab^	4.653 × 10^4^	6.350 × 10^6^	9.536 × 10^8 a^	7.571 × 10^6^	15.221 × 10^10^
Fourth instar/pupae	204	00.26 (3)	4.07 ± 0.76	5.920 × 10^5 b^	5.232 × 10^5^	6.700 × 10^6^	11.383 × 10^8 a^	9.099 × 10^7^	19.937 × 10^10^

^†^—Number of insect used for bioassay study; **^‡^**—Degree of freedom. *—LC_50_ of one treatment is significantly different if the both lower and upper fiducial limit does not include LC_50_ value of other treatments; the LC_50_ values are expressed as concentration of conidia mL^−1^. Different letters in the superscript denotes significant difference between treatments.

**Table 2:** Time—dose toxicity of *S. lanosoniveum* against different developmental stages of *A. rugioperculatus* at the concentration of 1 × 10^8^ conidia per mL.

Developmental Stages of RSW	n ^†^	X^2^ (DF) ^‡^	Slope	LT_50_ *	LT_50_ Confidence Interval	
					Lower	Upper
Egg	165	1.523 (4)	5.54 ± 0.38	4.27 ^a^	4.09	4.45
First instar	43	0.565 (4)	7.67 ± 0.91	4.86 ^b^	4.57	5.16
Second-third instar	63	2.406 (4)	5.16 ± 0.59	4.56 ^ab^	4.23	4.88
Fourth instar/pupae	35	2.680 (4)	10.4 ± 1.29	5.89 ^c^	5.59	6.20

^†^—Number of insect used for bioassay study; **^‡^**—Degree of freedom. *—LT_50_ of one treatment is significantly different if the both lower and upper fiducial limit does not include LT_50_ value of other treatments; the LT_50_ values are expressed as time taken in terms of days (d) to kill 50% population. Different letters in the superscript denotes significant difference between treatments.

## Data Availability

The data presented in this study are available on request from the corresponding author.
